# Supplementary material for: Genetic and neuronal mechanisms governing the sex-specific interaction between sleep and sexual behaviors in Drosophila
Source: Nat Commun. 2017 Jul 28;8:154. doi: 10.1038/s41467-017-00087-5 (PMC5533705; doi:10.1038/s41467-017-00087-5)
Supplement: Supplementary file 1 — Supplementary Information [file 41467_2017_87_MOESM1_ESM.pdf]

File Name: Supplementary Information
Description: Supplementary Figures

File Name: Peer Review File
Description:

File Name: Supplementary Movie 1
Description: Registration of P1 and DN1 neurons in a standard brain.

File Name: Supplementary Movie 2
Description: Calcium response of DN1 neurons after applying ATP to P2X2-expressing P1 neurons in R18H11-LexA/LexAop2-GCamp6m; R15A01-GAL4/UAS-P2X2 males.

File Name: Supplementary Movie 3
Description: Calcium response of DN1 neurons after applying ATP to P1 neurons without P2X2 in R18H11-LexA/LexAop2-GCamp6m; R15A01-GAL4/+ males.

File Name: Supplementary Movie 4
Description: Calcium response of P1 neurons after applying ATP to DN1 neurons (starts from 6s of the movie) in R18H11-LexA/LexAop2- P2X2; R15A01-GAL4/UAS-GCamp6m males.

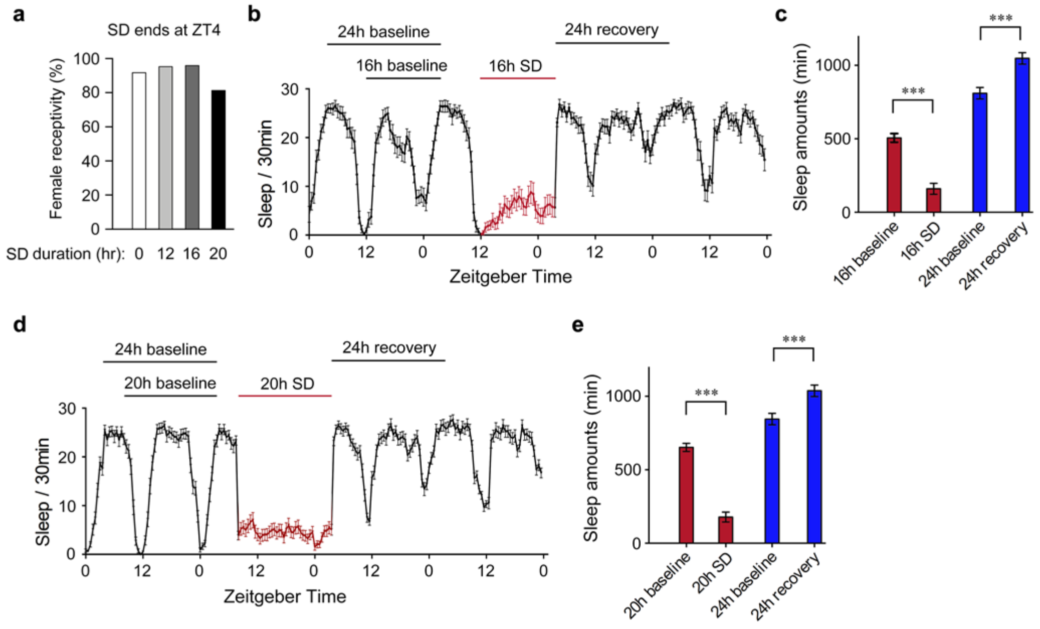

Supplementary Figure 1. Sleep deprivation does not affect female receptivity in wild-type flies. (**a**) Receptivity of females that had been sleep deprived for 12 hours (light gray), 16 hours (dark gray) and 20 hours (dark) during indicated periods. $n = 48$ for each. (**b**) Detailed sleep profile of 16-hour SD (15s/min shaking) in female flies. (**c**) The 16-hour SD results in 346-min sleep loss during SD, and induces post-SD sleep rebound. $n = 32$. ***$p < 0.001$, unpaired t-test. (**d**) Detailed sleep profile of 20-hour SD (15s/min shaking) in female flies. (**e**) The 20-hour SD results in 470-min sleep loss during SD, which is equivalent to 16-hour sleep loss in males (see Fig. 1c), and induces post-SD sleep rebound. $n = 32$. ***$p < 0.001$, unpaired t-test. Error bars indicate SEM.

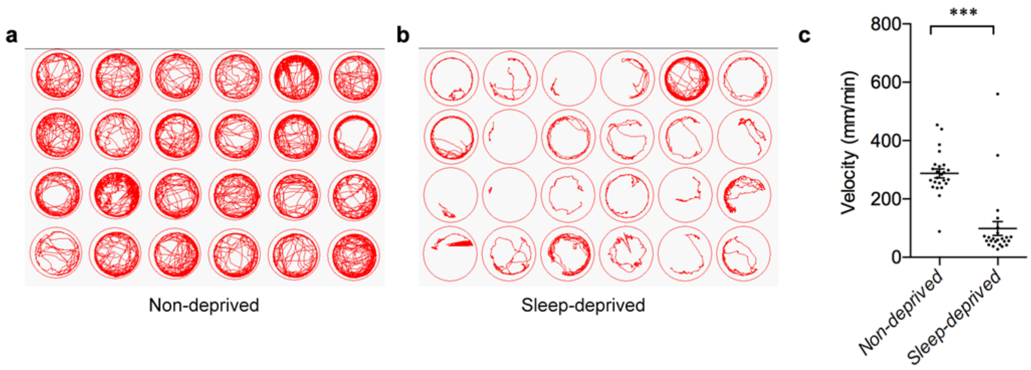

Supplementary Figure 2. Locomotor activity of males after sleep deprivation. Males were sleep deprived for 16 hours, and allowed for 30-min rest, then tested individually for 10min. (**a**) 10-min trajectories of non-deprived males. (**b**) 10-min trajectories of sleep-deprived males. (**c**) Mean velocity of sleep-deprived and non-deprived males. n = 24 for each. ***p < 0.001, unpaired t-test.

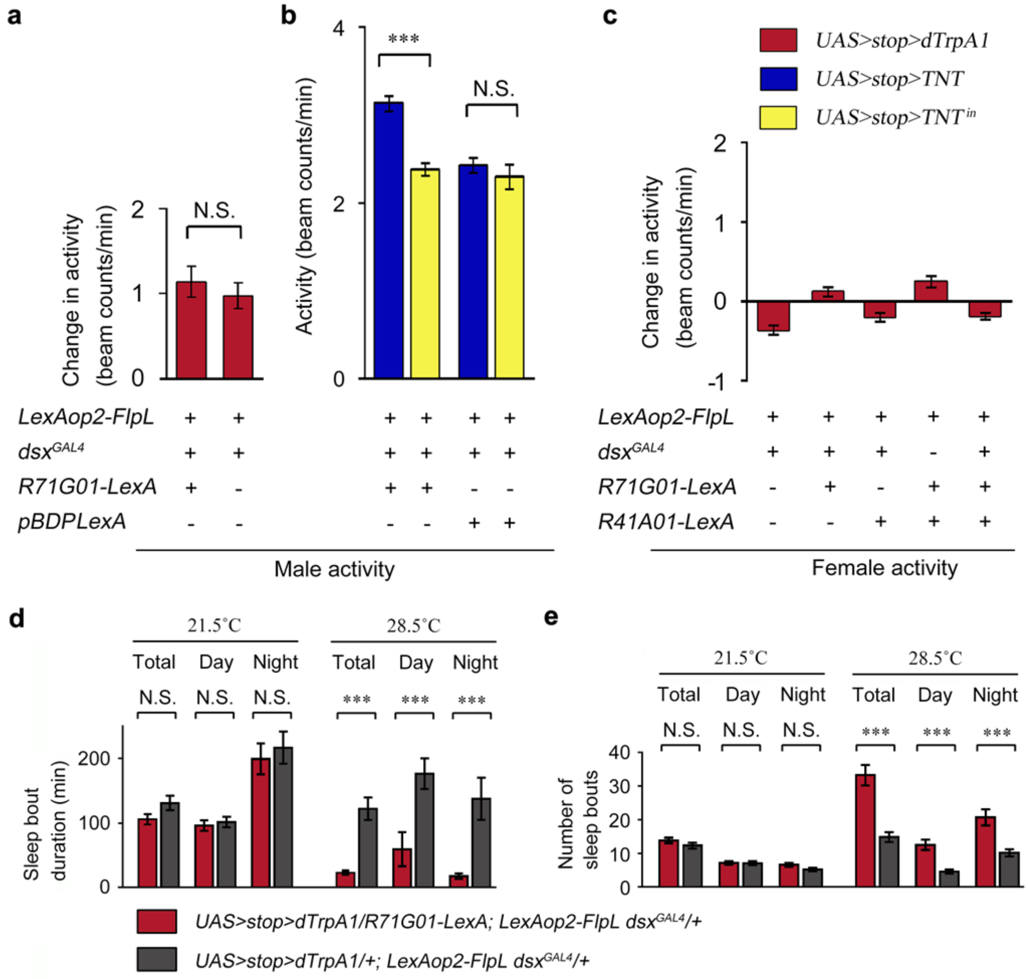

Supplementary Figure 3. P1-activated males have decreased sleep bout duration and increased number of sleep bouts. (**a**) Activity change from 21.5˚C to 28.5˚C is not different in control and experimental males. (**b**) Blocking neuronal transmission from P1 neurons increases male sleep (Fig. 2b), which is not due to activity decrease, as the activity actually increases. (**c**) Activity change from 21.5˚C to 28.5˚C is subtle in all 5 genotypes that specifically activate pC1 and/or pCd neurons. (**d** and **e**) Sleep bout duration (**d**) and number of sleep bouts (**e**) at 21.5˚C and 28.5˚C in total time, daytime and nighttime. Genotypes as indicated. n = 24~32 for each. ***$p < 0.001$, unpaired t-test. N.S., not significant. Error bars indicate SEM.

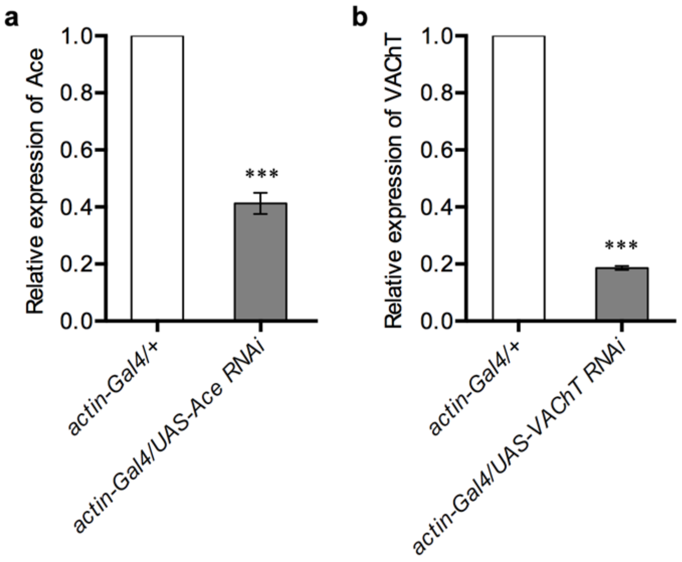

Supplementary Figure 4. Validation of RNAi efficiency. (**a** and **b**) Relative expression of Ace (**a**) and VAChT (**b**) are significantly reduced in RNAi-knockdown flies. Flies with Ace knock-down are fully viable, while flies with VAChT knock-down are partially lethal.

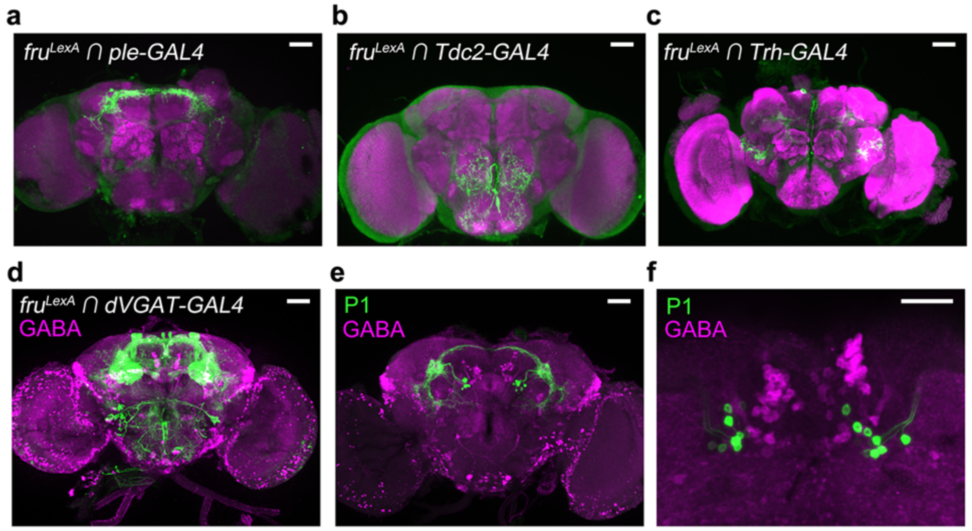

Supplementary Figure 5. *fru^{M}* overlaps with selected neurotransmitters in specific neurons, but not P1. (**a-d**) Intersectional expression between *fru^{LexA}* and *ple-GAL4* (**a**), *Tdc2-GAL4* (**b**), *Trh-GAL4* (**c**) and *dVGAT-GAL4* (**d**). (**e** and **f**) There is no anti-GABA signal in P1 neurons. Scale bars, 50μm.

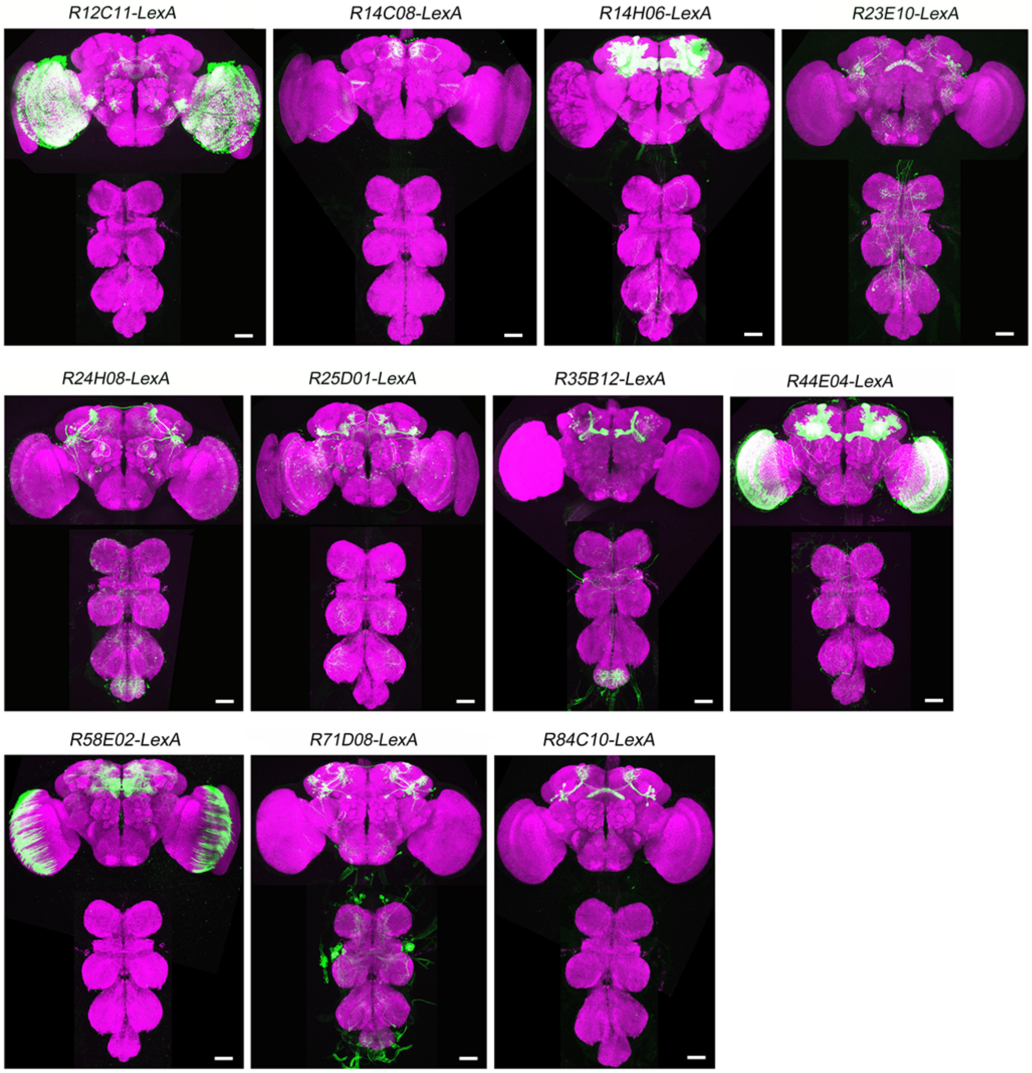

Supplementary Figure 6. Expression pattern of selected *LexA* lines in the central nervous system. Scale bars, 50μm.

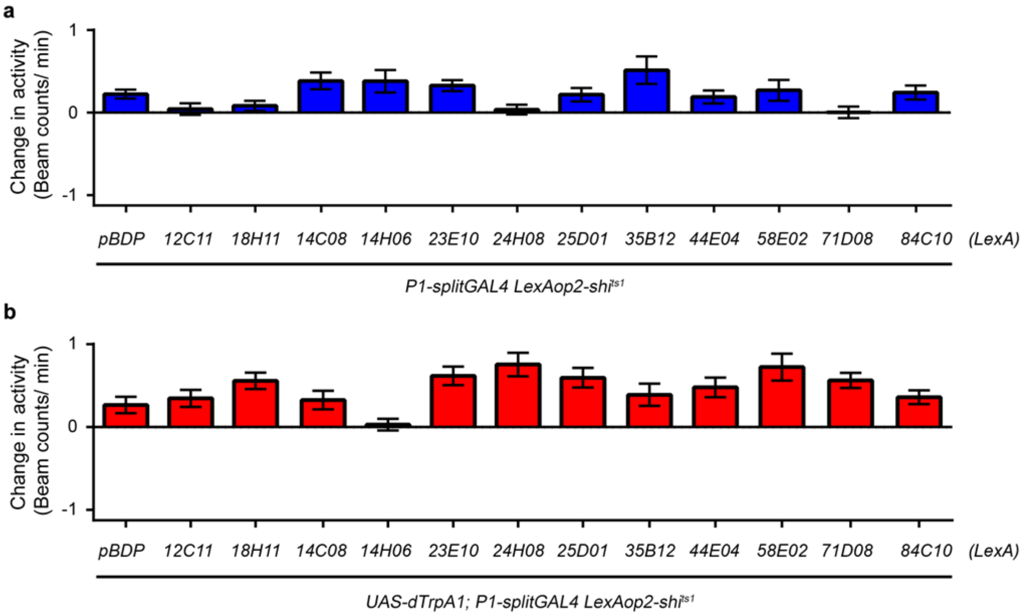

Supplementary Figure 7. The partial rescue of sleep deficit in P1-activated males by inhibiting DN1 neurons is not due to changes in general locomotor activity. (**a**) Activity changes from 21˚C to 29.5˚C when inhibiting *LexA*-labeled neurons. (**b**) Activity changes from 21˚C to 29.5˚C when inhibiting *LexA*-labeled neurons in addition to activating P1 neurons. *n* = 24~56 for each. Genotypes as indicated. Error bars indicate SEM.

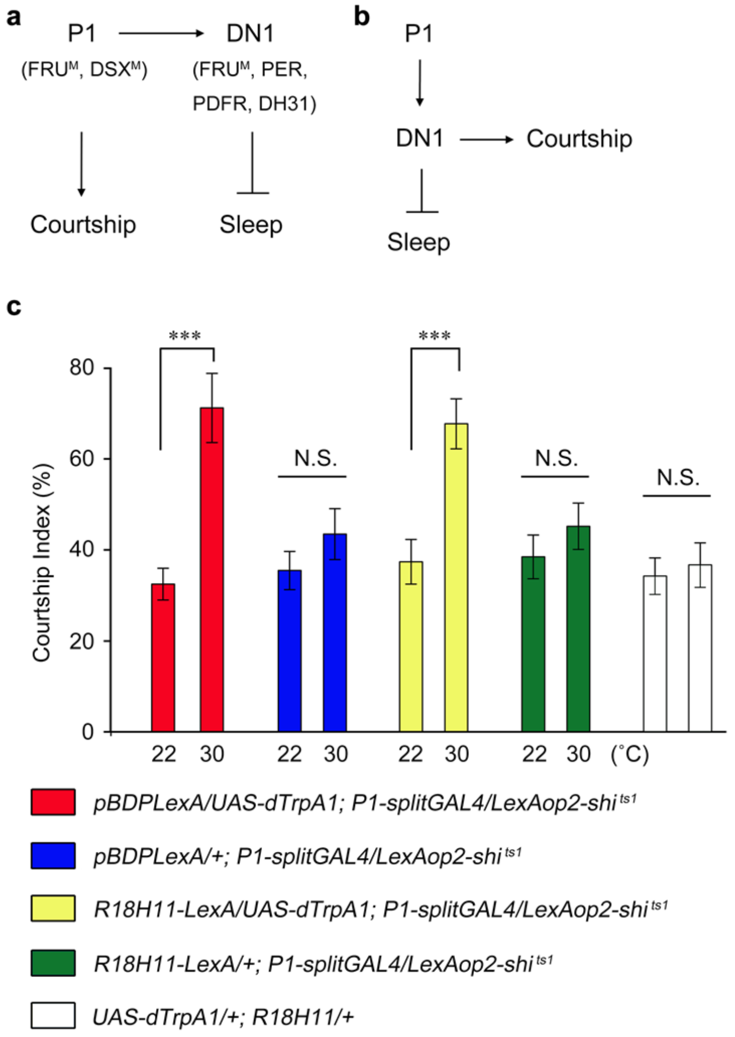

Supplementary Figure 8. Promotion of male courtship behavior by P1 neurons is independent of DN1 neurons. (**a** and **b**) Two models of the role of P1 and DN1 neurons in regulating sex and sleep. (**c**) Courtship indices of indicated genotypes in typical 10-min courtship assays. Red bars: activating P1 neurons alone promotes male courtship behavior to virgin headless females; blue bars: control flies without *dTrpA1* showed similar levels of courtship at 22˚C and 30˚C; yellow bars: activating P1 neurons in addition to inhibiting DN1 neurons promotes male courtship, which is similar with P1 activation alone; green bars: inhibiting DN1 neurons does not affect male courtship; white bars: activating DN1 neurons does not affect male courtship. n = 24 for each. ***$p < 0.001$, unpaired t-test. N.S., not significant. Error bars indicate

SEM. These results favor model A that P1 neurons promote male courtship independent of DN1 neurons.

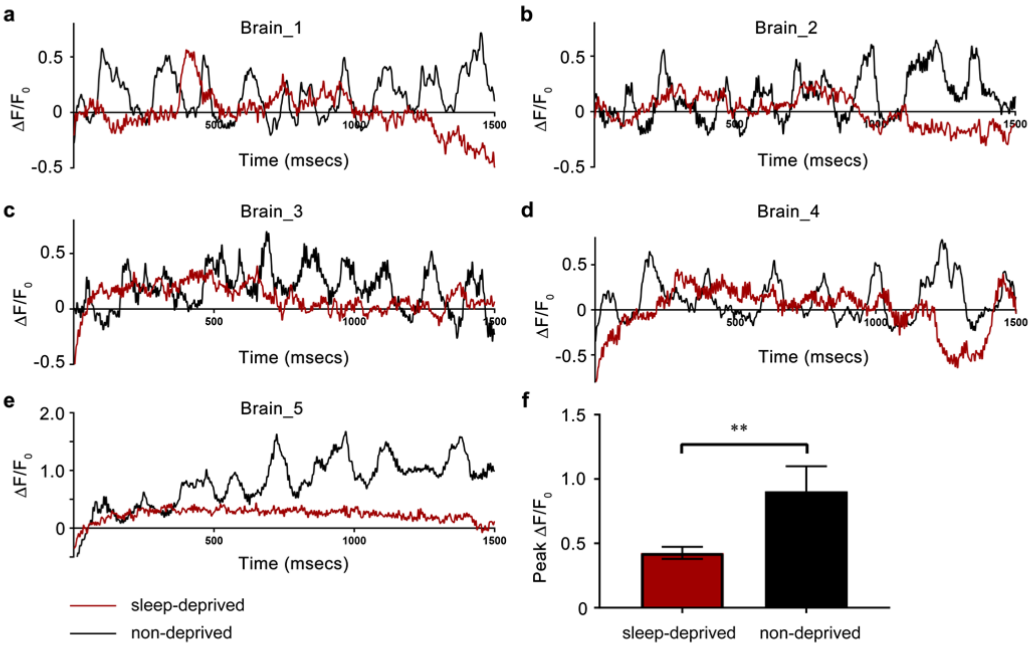

Supplementary Figure 9. Sleep deprivation decreases activity in P1 neurons. (**a-e**) Representative samples of fluorescent changes ($\Delta F/F_0$) of P1 neurons in sleep-deprived and non-deprived males. (f) Peak fluorescence changes ($\Delta F/F_0$) of P1 neurons. n = 10 for each, **$p < 0.01$, Unpaired t-test. Error bars indicate SEM.

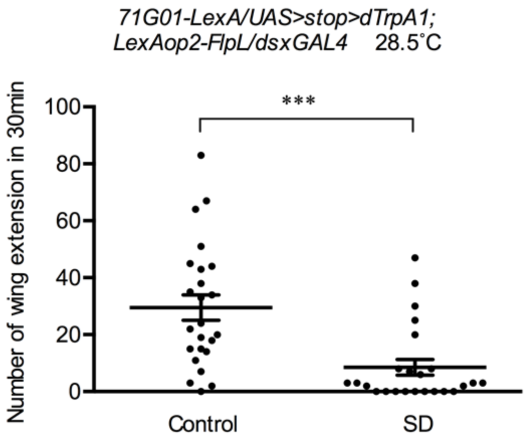

Supplementary Figure 10. Sleep deprivation (SD) inhibits P1-induced wing extension. Males were sleep-deprived for 16 hours at 21.5˚C, then transferred to 28.5˚C to activate P1 neurons and assay wing extension for 30 min. *n* = 24 for each, ***$p < 0.001$, Unpaired t-test.

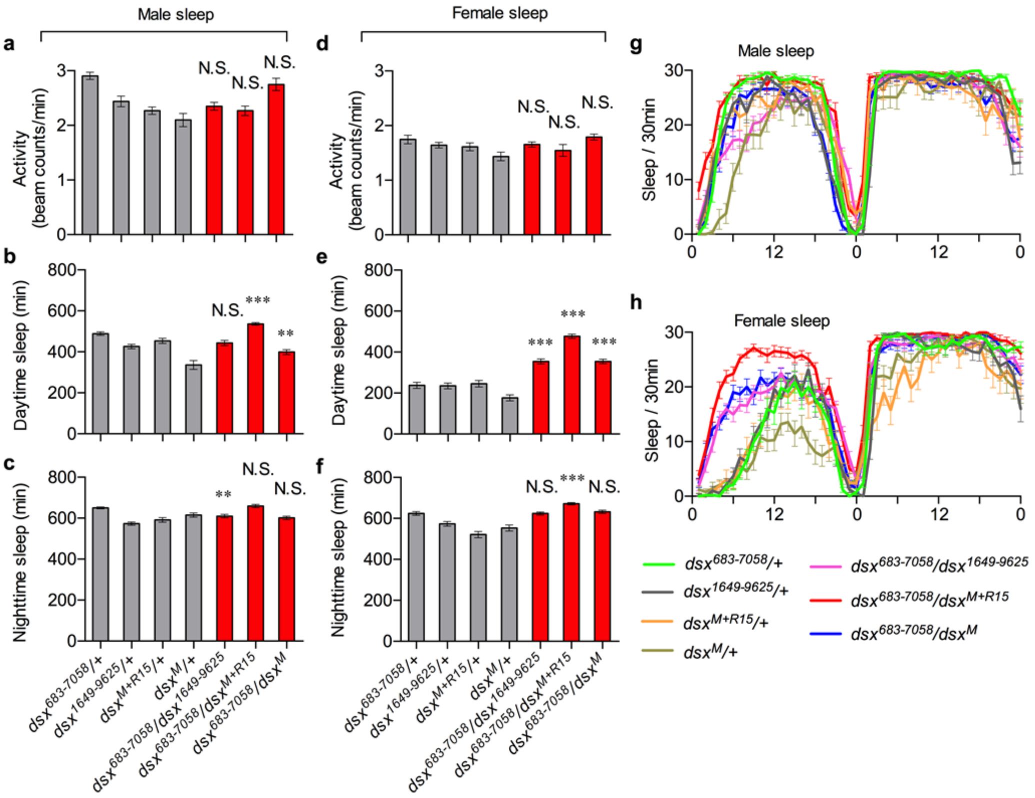

Supplementary Figure 11. DSX^{F} inhibits specifically daytime sleep in females. (**a-c**) Waking activity (**a**), daytime sleep (**b**) and nighttime sleep (**c**) of males with indicated genotypes. (**d-f**) Waking activity (**d**), daytime sleep (**e**) and nighttime sleep (**f**) of females with indicated genotypes. *n* = 24~32 for each. $^{**}p < 0.01$, $^{***}p < 0.001$, comparisons are made between the genotype and its parental genotypes, one-way ANOVA. N.S., not significant. **(g** and **h**) Detailed sleep profiles of male (**g**) and female (**h**) *dsx* alleles. Error bars indicate SEM.

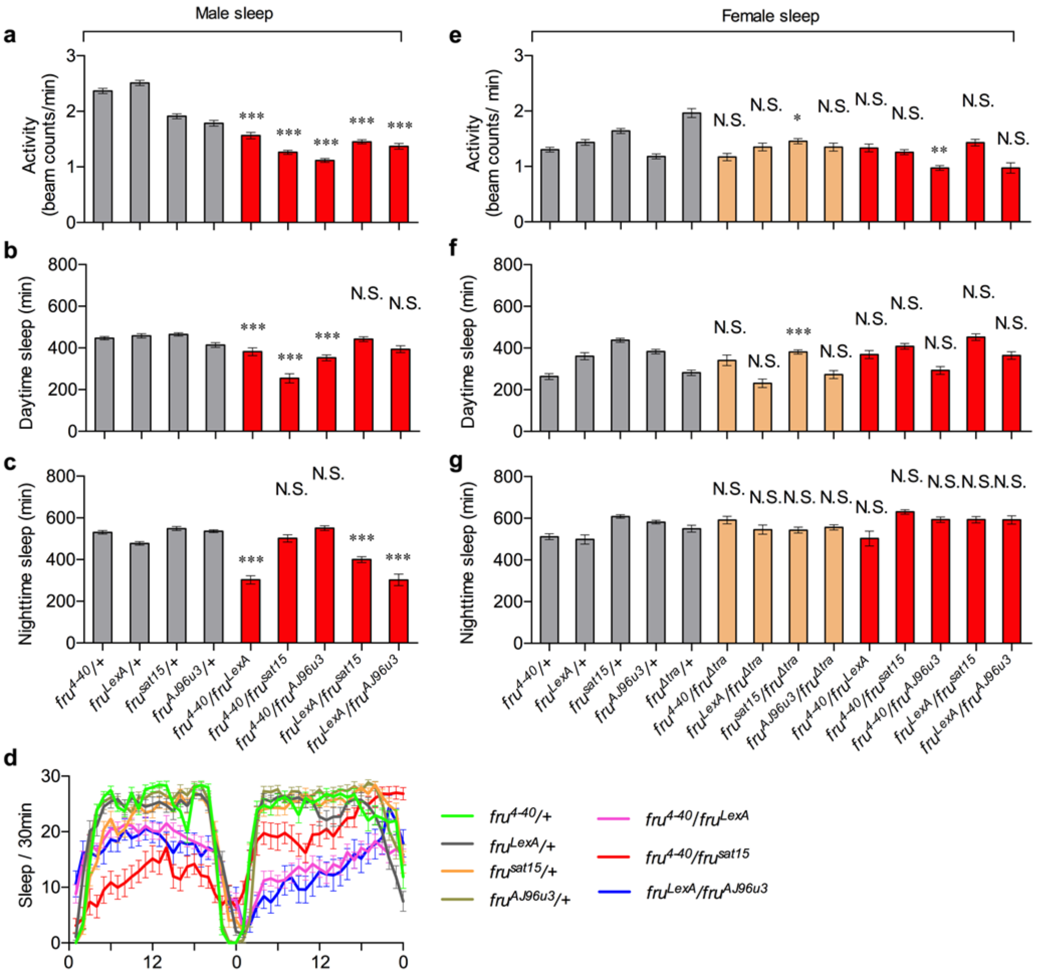

Supplementary Figure 12. FRU^{M} promotes both daytime and nighttime sleep in males. (**a-c**) Waking activity (**a**), daytime sleep (**b**) and nighttime sleep (**c**) of males with indicated genotypes. (**d**) Detailed sleep profiles of male flies with *fru*^{M} null combinations. (**e-g**) Waking activity (**e**), daytime sleep (**f**) and nighttime sleep (**g**) of females with indicated genotypes. n = 24~32 for each. *p < 0.05, **p < 0.01, ***p < 0.001, comparisons are made between the genotype and its parental genotypes, one-way ANOVA. N.S., not significant. Error bars indicate SEM.

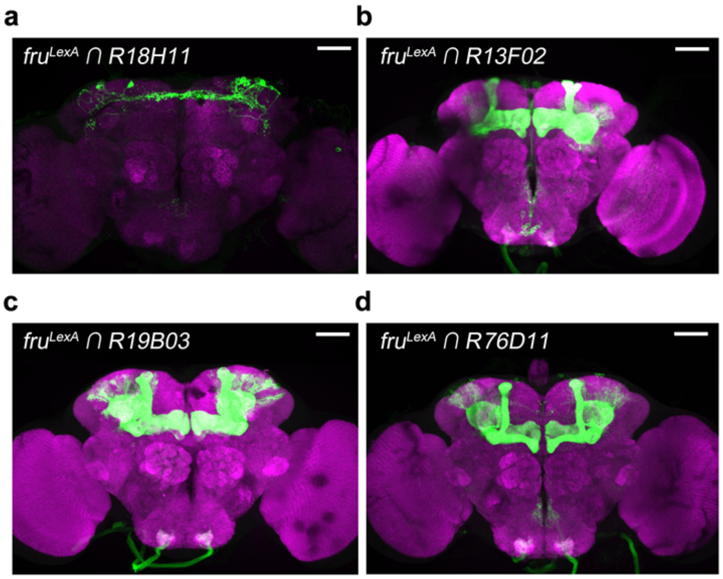

Supplementary Figure 13. MB and DN1 neurons are *fru*^{M}-positive. (**a**-**d**) Intersectional expression between *fru*^{LexA} and *R18H11* (**a**), *R13F02* (**b**), *R19B03* (**c**) and *R76D11* (**d**) in male brains. Scale bars, 50µm.

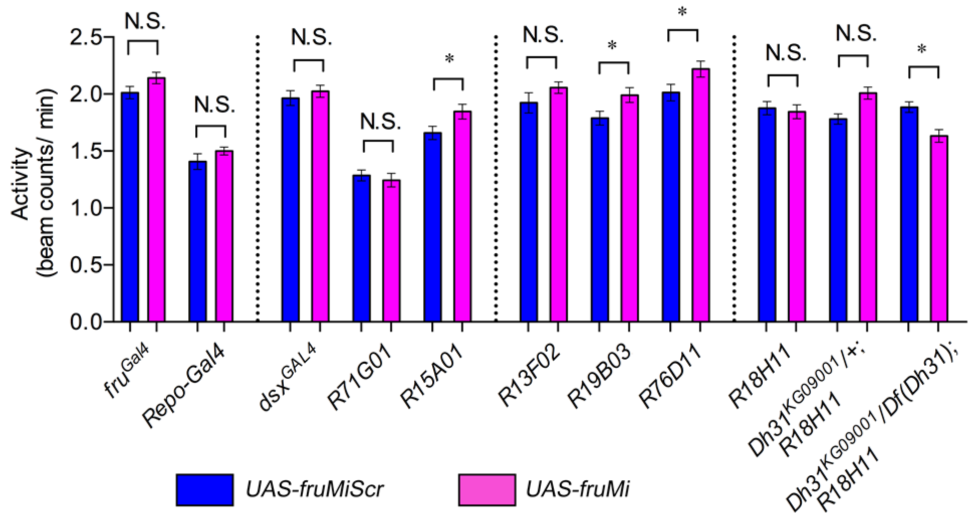

Supplementary Figure 14. Promotion of male sleep by FRU^{M} in DN1 neurons is not due to changes in general locomotor activity. Genotypes as indicated. n = 24~32 for each. *$p < 0.05$, unpaired t-test. N.S., not significant. Error bars indicate SEM.
